# The chromosomal genome sequence of an acoel, *Convolutriloba macropyga* Shannon & Achatz, 2007 (Acoela: Convolutidae)

**DOI:** 10.12688/wellcomeopenres.27384.1

**Published:** 2026-08-20

**Authors:** Francesca Pinton, Andreas Hejnol, Jose Victor Lopez, Nina Pruzinsky, Graeme Oatley, Elizabeth Sinclair, Eerik Aunin, Noah Gettle, Camilla Santos, Michael Paulini, Haoyu Niu, Victoria McKenna, Rebecca O’Brien, Wellcome Sanger Institute Tree of Life Management, Samples and Laboratory team, Wellcome Sanger Institute Scientific Operations: Sequencing Operations, Wellcome Sanger Institute Tree of Life Core Informatics team, EBI Aquatic Symbiosis Genomics Data Portal Team, Aquatic Symbiosis Genomics Project Leadership

**Affiliations:** 1 Institute of Zoology and Evolutionary Research, Friedrich Schiller University Jena, Jena, Germany; 2 Department of Biological Sciences, University of Bergen, Bergen, Norway; 3 Department of Biological Sciences, Nova Southeastern University, Dania Beach, Florida, USA; 4 NOAA University Corporation for Atmospheric Research, Boulder, Colorado, USA; 5 NOAA Ocean Exploration Affiliate, Silver Spring, Maryland, USA; 6 Tree of Life Programme, Wellcome Sanger Institute, Hinxton, England, UK

**Keywords:** Convolutriloba macropyga, acoel, genome sequence, chromosomal, Acoela

## Abstract

We present a genome assembly from a specimen of *Convolutriloba macropyga* (acoel; Xenacoelomorpha; Acoela; Convolutidae). The genome sequence has a total length of 1 175.98 megabases. Most of the assembly (88.04%) is scaffolded into 15 chromosomal pseudomolecules. The mitochondrial genome has also been assembled, with a length of 15.47 kilobases.

## Species taxonomy

Eukaryota; Opisthokonta; Metazoa; Eumetazoa; Bilateria; Xenacoelomorpha; Acoelomorpha; Acoela; Convolutidae; *Convolutriloba*; *Convolutriloba macropyga* ( Shannon & Achatz, 2007) (NCBI:txid536237).

## Background

*Convolutriloba macropyga* ( Shannon & Achatz, 2007) is a soft-bodied marine invertebrate belonging to Convolutidae (Acoela, Xenacoelomorpha). Because of their key phylogenetic position as the likely sister group to all other bilaterians, Xenacoelomorpha are highly relevant to evolutionary studies, for example, to clarify the origin of the through-gut and nerve-cord organisation ( Andrikou *et al.*, 2025; Hejnol & Martindale, 2008; Hejnol & Pang, 2016). Acoela are the most species-rich and diverse xenacoelomorph group, with Convolutidae showing the highest molecular evolutionary rates ( Abalde & Jondelius, 2024; Jondelius *et al.*, 2019). Among Convolutidae, symbiosis with photosynthetic microbes (photosymbiosis) has evolved at least twice and can involve dinoflagellates, green algae or diatoms ( Achatz *et al.*, 2010; Pinton *et al.*, 2026; Riewluang *et al.*, 2026).

While the geographical distribution of *C. macropyga* is not known, its optimal culture conditions suggest tropical environments ( Shannon & Achatz, 2007). Individuals of *C. macropyga* are less than 10 mm long and dorsoventrally flattened. The anterior end is rounded, whereas three or more lobes can be observed at the posterior end ( Figure 1). The mouth – the only opening to the digestive system – is located in the middle of the ventral side, while the nervous system is composed of longitudinal nerve cords running from anterior ganglia associated with colourless eyespots ( Shannon & Achatz, 2007). The animals’ green colour, caused by symbiotic green algae distributed throughout the body, is interspersed with gland cells containing red-orange rhabdoids and sagittocysts, while red pigment cells form a diamond-shaped pattern in the posterior region ( Shannon & Achatz, 2007). Individuals are simultaneous hermaphrodites, with paired testes and ovaries in the middle of the body. Like all other *Convolutriloba* species, they can also reproduce asexually: new individuals bud from the posterior end of the body with an axis reversed relative to that of the parent ( Shannon & Achatz, 2007; Sikes & Bely, 2008).

**Figure 1.**
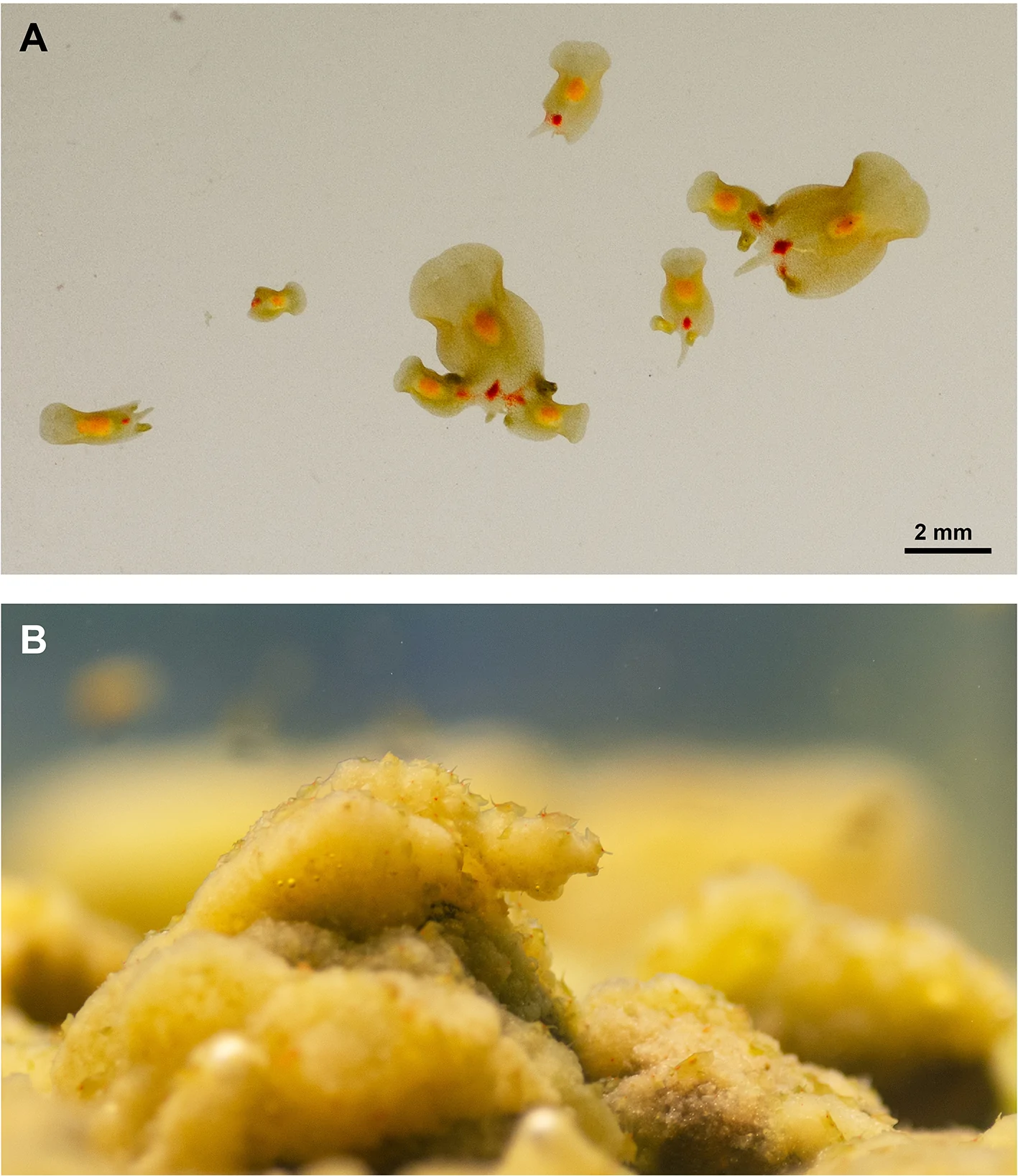
Photographs of *Convolutriloba macropyga* ( Shannon & Achatz, 2007). (A) Ventral view of specimens of different sizes. The digestive system filled with food (orange) is visible in the middle of the body, as well as asexual offspring budding from the posterior side of the animal. (B) Animals on a substrate of algae in a culture aquarium.

This mode of asexual reproduction makes *Convolutriloba* species especially interesting, particularly *C. macropyga*, the most prolific species in the genus. Among photosymbiotic species, *Convolutriloba* species are the only ones that reproduce both sexually and asexually ( Åkesson *et al.*, 2001; Åkesson & Hendelberg, 1989; Shannon & Achatz, 2007). This affects the mode of symbiont transmission: vertical during asexual reproduction and horizontal following sexual reproduction ( Shannon & Achatz, 2007).

Photosymbiosis research in *Convolutriloba* species has so far described the location and ultrastructure of symbionts and shown that the symbiosis is obligate for the host and affected by temperature increases, injuries and infections ( Hirose & Hirose, 2007; Nanes Sarfati *et al.*, 2024; Pinton *et al.*, 2026; Querido *et al.*, 2025).

This high-quality genome of *C. macropyga* will be an important resource for understanding the morphological diversification of Convolutidae ( Abalde & Jondelius, 2024). Moreover, it will allow comparisons with other photosymbiotic species, helping to clarify genomic adaptations to photosymbiosis in Acoela and Metazoa ( Melo Clavijo *et al.*, 2018).

## Methods

### Sample acquisition

The specimen used for genome sequencing was an adult H *Convolutriloba macropyga* (specimen ID NSU0040706, ToLID txConMacr16; Figure 1), collected from Bergen Animal Culture, Norway (latitude 60.3813, longitude 5.3325) on 2020-11-05. A second specimen was used for Hi-C sequencing (specimen ID NSU0040707, ToLID txConMacr17), and a third specimen was used for RNA sequencing (specimen ID NSU0040704, ToLID txConMacr14). It was collected from Bergen Animal Culture, Norway (latitude 60.3813, longitude 5.3325) on 2020-11-05. The specimens wrtr collected by Andreas Hejnol (University of Bergen) and identified by Tom Shannon and Johannes Achatz (University of Innsbruck). Animals were kept in culture in in filtered sea water with salinity 36‰ at 26 °C with a cycle of 10 h darkness: 14 h light, and they were fed twice a week with brine shrimp *Artemia.*

### Nucleic acid extraction

Protocols for high molecular weight (HMW) DNA extraction developed at the Wellcome Sanger Institute (WSI) Tree of Life Core Laboratory are available on protocols.io ( Howard *et al.*, 2025). The txConMacr16 sample was weighed and triaged to determine the appropriate extraction protocol. Tissue from the whole organism was homogenised by powermashing using a PowerMasher II tissue disruptor. HMW DNA was extracted using the Manual MagAttract v3 protocol. We used centrifuge-mediated fragmentation to produce DNA fragments in the 8–10 kb range, following the Covaris g-TUBE protocol for ultra-low input (ULI). Sheared DNA was purified by automated SPRI (solid-phase reversible immobilisation). sheared and purified DNA was assessed using a Nanodrop spectrophotometer and Qubit Fluorometer using the Qubit dsDNA High Sensitivity Assay kit. Fragment size distribution was evaluated by running the sample on the FemtoPulse system. For this sample, the final post-shearing DNA had a Qubit concentration of 0.562 ng/μL and a fragment size of 10.2 kb.

RNA was extracted from whole organism tissue of the txConMacr14 specimen in the Tree of Life Laboratory at the WSI using the RNA Extraction: Automated MagMax™ *mir*Vana protocol. The RNA concentration was assessed using a Nanodrop spectrophotometer and a Qubit Fluorometer using the Qubit RNA Broad-Range Assay kit. Analysis of the integrity of the RNA was done using the Agilent RNA 6000 Pico Kit and Eukaryotic Total RNA assay.

### PacBio HiFi library preparation and sequencing

Library preparation and sequencing were performed at the WSI Scientific Operations core. Prior to library preparation, the DNA was fragmented to ~10 kb. Ultra-low-input (ULI) libraries were prepared using the PacBio SMRTbell ^®^ Express Template Prep Kit 2.0 and gDNA Sample Amplification Kit. Samples were normalised to 20 ng DNA. Single-strand overhang removal, DNA damage repair, and end-repair/A-tailing were performed according to the manufacturer’s instructions, followed by adapter ligation. A 0.85× pre-PCR clean-up was carried out with Promega ProNex beads.

The DNA was evenly divided into two aliquots for dual PCR (reactions A and B), both following the manufacturer’s protocol. A 0.85× post-PCR clean-up was performed with ProNex beads. DNA concentration was measured using a Qubit Fluorometer v4.0 (Thermo Fisher Scientific) with the Qubit HS Assay Kit, and fragment size was assessed on an Agilent Femto Pulse Automated Pulsed Field CE Instrument (Agilent Technologies) using the gDNA 55 kb BAC analysis kit. PCR reactions A and B were then pooled, ensuring a total mass of ≥500 ng in 47.4 μl.

The pooled sample underwent another round of DNA damage repair, end-repair/A-tailing, and hairpin adapter ligation. A 1× clean-up was performed with ProNex beads, followed by DNA quantification using the Qubit and fragment size analysis using the Agilent Femto Pulse. Size selection was performed on the Sage Sciences PippinHT system, with target fragment size determined by Femto Pulse analysis (typically 4–9 kb). Size-selected libraries were cleaned with 1.0× ProNex beads and normalised to 2 nM before sequencing.

The sample was sequenced on a Revio instrument (Pacific Biosciences, California, USA). Prepared libraries with a molarity greater than 2 nM were normalised to 2 nM, and 15 μL was used for making complexes. For libraries with a molarity below 2 nM, the available 10 μL library volume was used without normalisation. Primers were annealed and polymerases were bound to generate circularised complexes, following the manufacturer’s instructions. Complexes were purified using 1.2× SMRTbell beads, diluted to the Revio loading concentration of 200–300 pM, and spiked with a Revio sequencing internal control. The sample was sequenced on a Revio 25M SMRT cell. SMRT Link software (Pacific Biosciences), a web-based workflow manager, was used to configure and monitor the run and to carry out primary and secondary data analysis.

### Hi-C

***Sample preparation and crosslinking***

The Hi-C sample was prepared from 20–50 mg of frozen whole organism tissue of the txConMacr17 sample using the Arima-HiC v2 kit (Arima Genomics). Following the manufacturer’s instructions, tissue was fixed and DNA crosslinked using TC buffer to a final formaldehyde concentration of 2%. The tissue was homogenised using the Diagnocine Power Masher-II. Crosslinked DNA was digested with a restriction enzyme master mix, biotinylated, and ligated. Clean-up was performed with SPRISelect beads before library preparation. DNA concentration was measured with the Qubit Fluorometer (Thermo Fisher Scientific) and Qubit HS Assay Kit. The biotinylation percentage was estimated using the Arima-HiC v2 QC beads.

***Hi-C library preparation and sequencing***

Biotinylated DNA constructs were fragmented using a Covaris E220 sonicator and size selected to 400–600 bp using SPRISelect beads. DNA was enriched with Arima-HiC v2 kit Enrichment beads. End repair, A-tailing, and adapter ligation were carried out with the NEBNext Ultra II DNA Library Prep Kit (New England Biolabs), following a modified protocol where library preparation occurs while DNA remains bound to the Enrichment beads. Library amplification was performed using KAPA HiFi HotStart mix and a custom Unique Dual Index (UDI) barcode set (Integrated DNA Technologies). Depending on sample concentration and biotinylation percentage determined at the crosslinking stage, libraries were amplified with 10–16 PCR cycles. Post-PCR clean-up was performed with SPRISelect beads. Libraries were quantified using the AccuClear Ultra High Sensitivity dsDNA Standards Assay Kit (Biotium) and a FLUOstar Omega plate reader (BMG Labtech).

Prior to sequencing, libraries were normalised to 10 ng/μL. Normalised libraries were quantified again to create equimolar and/or weighted 2.8 nM pools. Pool concentrations were checked using the Agilent 4200 TapeStation (Agilent) with High Sensitivity D500 reagents before sequencing. Sequencing was performed using paired-end 150 bp reads on the Illumina NovaSeq X.

### RNA library preparation and sequencing

Libraries were prepared using the NEBNext® Ultra II Directional RNA Library Prep Kit for Illumina (New England Biolabs), following the manufacturer’s instructions. Poly(A) mRNA was isolated from 100 ng of total RNA using oligo (dT) beads, converted to cDNA, and uniquely indexed; 14 PCR cycles were performed. Libraries were prepared with an intended fragment size of 100–300 bp. Libraries were quantified, normalised, and pooled equimolarly to a final concentration of 2.8 nM before dilution to 150 pM for loading. Sequencing was carried out on the Illumina NovaSeq X, generating paired-end reads.

### Genome assembly

Prior to assembly of the PacBio HiFi reads, a database of *k*-mer counts ( *k* = 31) was generated from the filtered reads using FastK. GenomeScope2 ( Ranallo-Benavidez *et al.*, 2020) was used to analyse the *k*-mer frequency distributions, providing estimates of genome size, heterozygosity, and repeat content.

The HiFi reads were assembled using Hifiasm ( Cheng *et al.*, 2021) with the --primary option. Haplotypic duplications were identified and removed using purge_dups ( Guan *et al.*, 2020). The Hi-C reads ( Rao *et al.*, 2014) were mapped to the primary contigs using bwa-mem2 ( Vasimuddin *et al.*, 2019), and the contigs were scaffolded in YaHS ( Zhou *et al.*, 2023) with the --break option for handling potential misassemblies. The scaffolded assemblies were evaluated using Gfastats ( Formenti *et al.*, 2022), BUSCO ( Manni *et al.*, 2021) and MerquryFK ( Rhie *et al.*, 2020).

The mitochondrial genome was assembled using MitoHiFi ( Uliano-Silva *et al.*, 2023). The MitoHiFi reference was the mitochondrial genome of *Symsagittifera roscoffensis* (NC_014578.1).

### Assembly curation

The assembly was decontaminated using the Assembly Screen for Cobionts and Contaminants ( ASCC) pipeline. TreeVal was used to generate the flat files and maps for use in curation. Manual curation was conducted primarily in PretextView and HiGlass ( Kerpedjiev *et al.*, 2018). Scaffolds were visually inspected and corrected as described by Howe *et al*. (2021). Manual corrections included 510 breaks, 798 joins, and removal of 224 haplotypic duplications. This reduced the scaffold count by 32.2%, increased the scaffold N50 by 5.5%, and reduced the total assembly length by 10.2%. The curation process is described at sanger-tol/curation-resources . PretextSnapshot was used to generate a Hi-C contact map of the final assembly.

### Assembly quality assessment

The Merqury.FK tool ( Rhie *et al.*, 2020) was run in a Singularity container ( Kurtzer *et al.*, 2017) to evaluate *k*-mer completeness and assembly quality for the primary and alternate haplotypes using the *k*-mer database ( *k* = 31) computed prior to genome assembly. The analysis outputs included assembly QV scores and completeness statistics.

The genome was analysed using the BlobToolKit pipeline, a Nextflow implementation of the earlier Snakemake version ( Challis *et al.*, 2020). PacBio reads were aligned to the assembly using minimap2 ( Li, 2018) and processed with SAMtools ( Danecek *et al.*, 2021) to generate coverage tracks. The pipeline runs BUSCO ( Manni *et al.*, 2021) using lineages identified from the NCBI Taxonomy ( Schoch *et al.*, 2020). For the three basal BUSCO lineages, eukaryota, bacteria and archaea, BUSCO genes are aligned to the UniProt Reference Proteomes database ( Bateman *et al.*, 2023) using DIAMOND BLASTp ( Buchfink *et al.*, 2021). The genome assembly is divided into chunks based on the density of BUSCO genes from the closest taxonomic lineage, and each chunk is aligned to the UniProt Reference Proteomes database using DIAMOND BLASTx. Sequences without DIAMOND BLASTx hits are extracted using seqtk and aligned to the NT database using BLASTn ( Altschul *et al.*, 1990). The outputs are consolidated into a BlobDir for visualisation in BlobToolKit. The BlobToolKit pipeline was developed using nf-core tooling ( Ewels *et al.*, 2020) and MultiQC ( Ewels *et al.*, 2016), with containerisation through Docker ( Merkel, 2014) and Singularity ( Kurtzer *et al.*, 2017).

## Genome sequence report

### Sequence data

PacBio sequencing of the *Convolutriloba macropyga* specimen generated 66.51 Gb (gigabases) from 8.21 million reads, which were used to assemble the genome. GenomeScope2.0 analysis using a *p* = 2 model gave a *k*-mer-based genome size estimate of 866.90 Mb, with a heterozygosity of 3.65% and repeat content of 40.01% ( Figure 2). These estimates guided expectations for the assembly. Based on the estimated genome size, the sequencing data provided approximately 64× coverage. Hi-C sequencing produced 110.19 Gb from 364.86 million reads, which were used to scaffold the assembly. RNA sequencing data were also generated and are available in public sequence repositories. Table 1 summarises the specimen and sequencing details.

**Figure 2.**
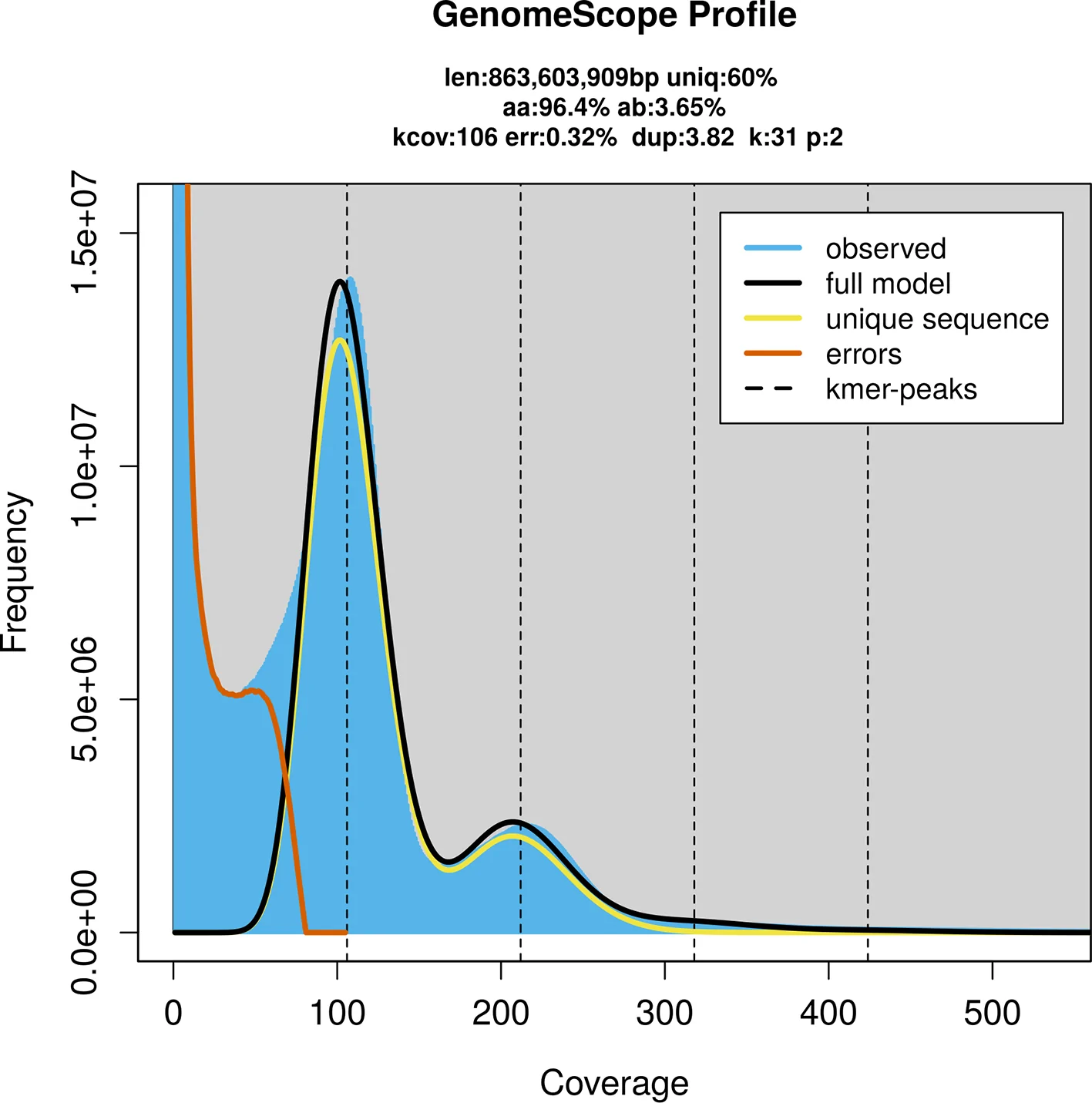
Frequency distribution of *k*-mers generated using GenomeScope2. The plot shows observed and modelled *k*-mer spectra, providing estimates of genome size, heterozygosity, and repeat content based on unassembled sequencing reads.

**Table 1.** Specimen and sequencing data for *Convolutriloba macropyga* (BioProject PRJEB75559).

Platform	PacBio HiFi	Hi-C	RNA-seq
**ToLID**	txConMacr16	txConMacr17	txConMacr14
**Specimen ID**	NSU0040706	NSU0040707	NSU0040704
**BioSample (source individual)**	SAMEA8576714	SAMEA8576715	SAMEA8576712
**BioSample (tissue)**	SAMEA8576724	SAMEA8576725	SAMEA8576722
**Tissue**	whole organism	whole organism	whole organism
**Instrument**	Revio	Illumina NovaSeq X	Illumina NovaSeq X
**Run accessions**	ERR13071481	ERR13093659	ERR13669978
**Read count total**	8.21 million reads	364.86 million read pairs	55.58 million read pairs
**Base count total**	66.51 Gb	110.19 Gb	16.79 Gb

### Assembly statistics

The primary haplotype was assembled, and contigs corresponding to an alternate haplotype were also deposited in INSDC databases. The final assembly has a total length of 1 175.98 Mb in 4 694 scaffolds, with 6 645 gaps, and a scaffold N50 of 68.62 Mb ( Table 2).

**Table 2.** Genome assembly data for *Convolutriloba macropyga.*

**Genome assembly**	Primary assembly
**Assembly name**	txConMacr16.3
**Assembly accession**	GCA_964194025.3
**Alternate haplotype accession**	GCA_964194055.3
**Assembly level**	chromosome
**Span (Mb)**	1 175.98
**Number of chromosomes**	15
**Number of contigs**	11 339
**Contig N50**	0.19 Mb
**Number of scaffolds**	4 694
**Scaffold N50**	68.62 Mb
**Organelles**	Mitochondrial genome: 15.47 kb

Most of the assembly sequence (88.04%) was assigned to 15 chromosomal-level scaffolds. These chromosome-level scaffolds, confirmed by Hi-C data, are named according to size ( Figure 3; Table 3). The exact order and orientation of the contigs on chromosome 15 (20.6–22.6 Mbp) are unknown.

**Figure 3.**
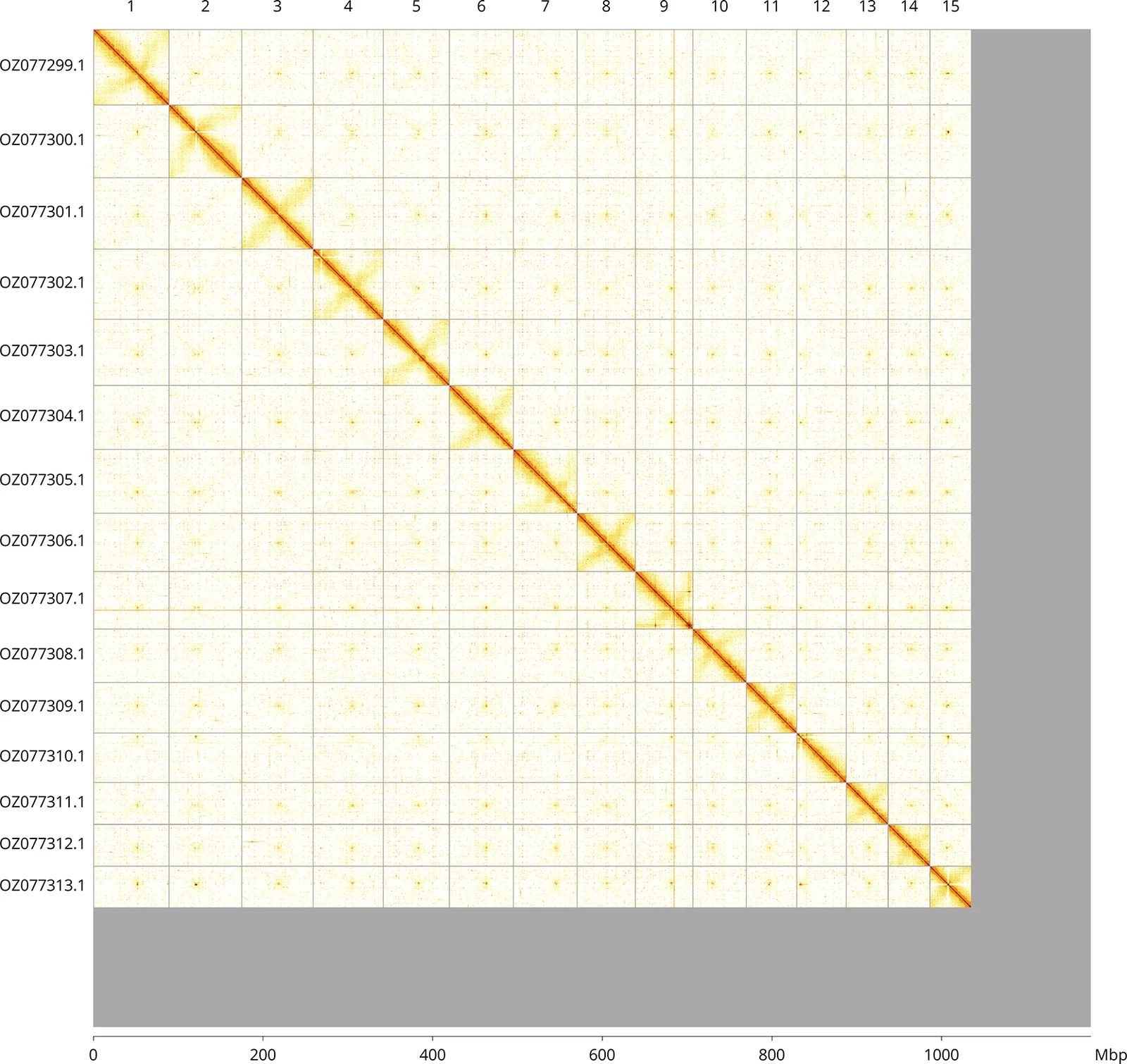
Hi-C contact map of the *Convolutriloba macropyga* genome assembly. Assembled chromosomes are shown in order of size and labelled along the axes, with a megabase scale shown below. The plot was generated using PretextSnapshot.

**Table 3.** Chromosomal pseudomolecules in the primary genome assembly of *Convolutriloba macropyga* txConMacr16.

INSDC accession	Molecule	Length (Mb)	GC%
OZ077299.1	1	89.26	35.50
OZ077300.1	2	85.96	35.50
OZ077301.1	3	83.90	35.50
OZ077302.1	4	82.96	35.50
OZ077303.1	5	77.66	35.50
OZ077304.1	6	75.67	35.50
OZ077305.1	7	75.07	35.50
OZ077306.1	8	68.62	35.50
OZ077307.1	9	67.91	35.50
OZ077308.1	10	62.79	35.50
OZ077309.1	11	59.72	35.50
OZ077310.1	12	58.42	35
OZ077311.1	13	49.48	35.50
OZ077312.1	14	49.04	35.50
OZ077313.1	15	48.88	35

The mitochondrial genome was also assembled (length 15.47 kb, OZ077314.1). This sequence is included as a contig in the multifasta file of the genome submission and as a standalone record.

### Assembly quality metrics

The combined primary and alternate assemblies achieve an estimated QV of 53.4. The *k*-mer completeness is 65.15% for the primary assembly, 56.09% for the alternate haplotype, and 97.62% for the combined assemblies ( Figure 4).

**Figure 4.**
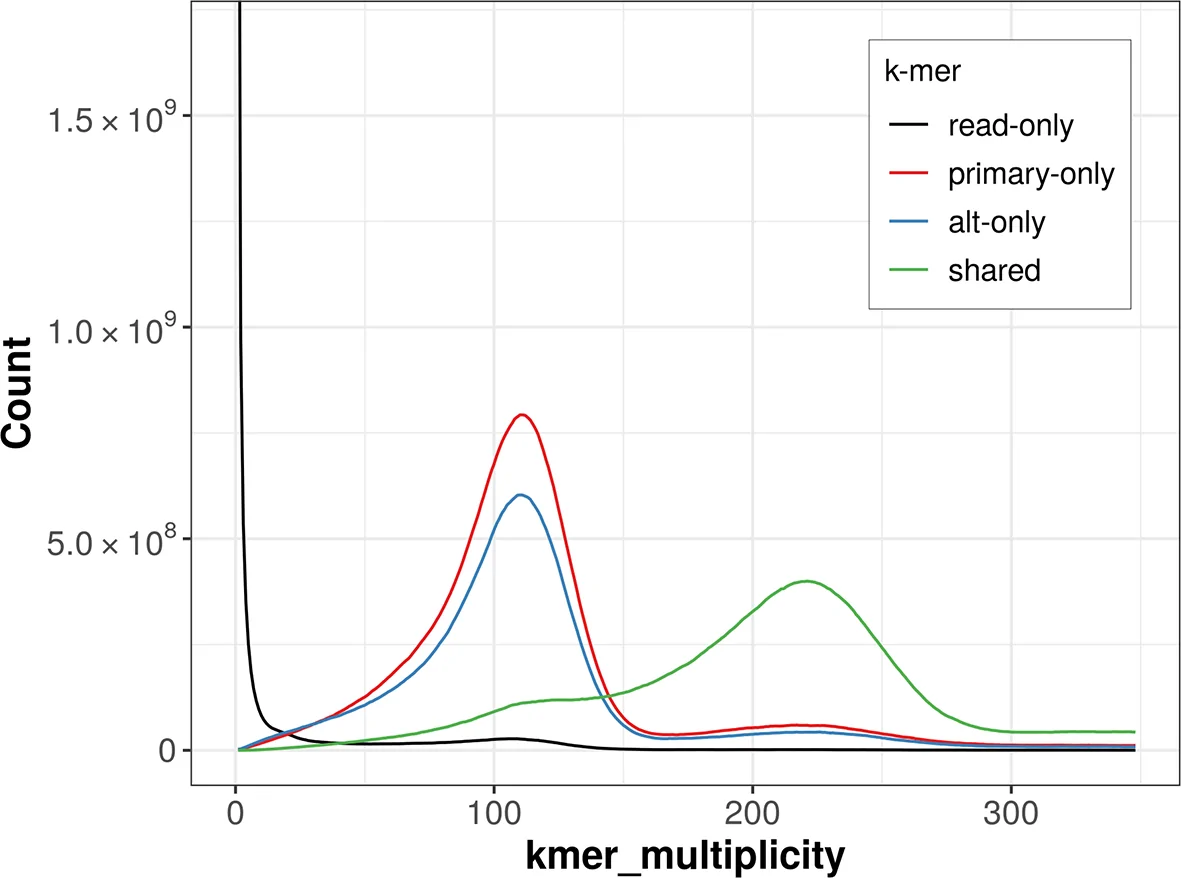
Evaluation of *k*-mer completeness using MerquryFK. This plot illustrates the recovery of *k*-mers from the original read data in the final assemblies. The horizontal axis represents *k*-mer multiplicity, and the vertical axis shows the number of *k*-mers. The black curve represents *k*-mers that appear in the reads but are not assembled. The green curve corresponds to *k*-mers shared by both haplotypes, and the red and blue curves show *k*-mers found only in one of the haplotypes.

BUSCO v.5.8.3 analysis using the metazoa_odb10 reference set ( *n* = 954) identified 69.0% of the expected gene set (single = 65.2%, duplicated = 3.8%). The snail plot in Figure 5 summarises the scaffold length distribution and other assembly statistics for the primary assembly. The blob plot in Figure 6 shows the distribution of scaffolds by GC proportion and coverage.

**Figure 5.**
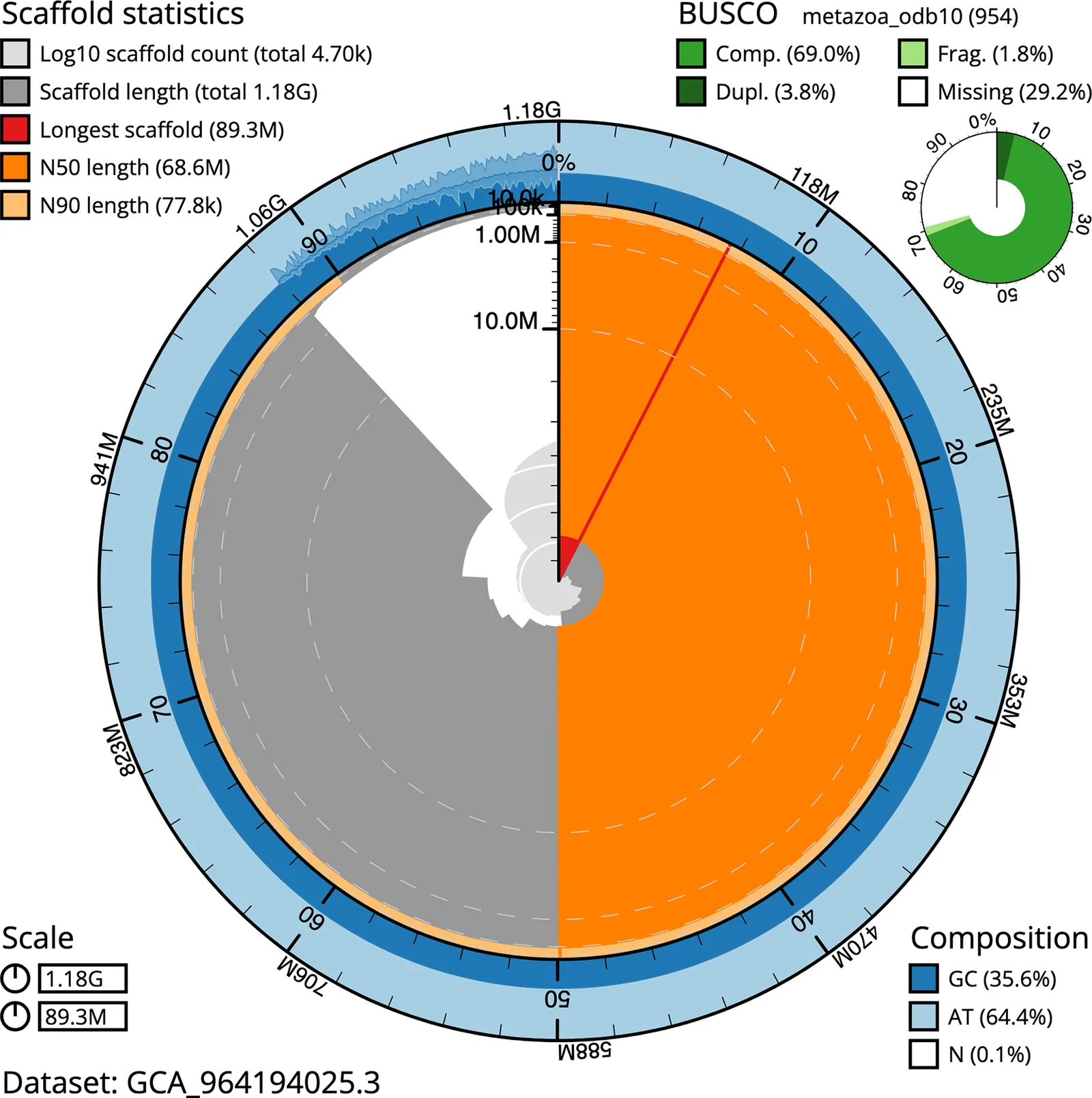
Assembly metrics for txConMacr16.3. The BlobToolKit snail plot provides an overview of assembly metrics and BUSCO gene completeness. The circumference represents the length of the whole genome sequence, and the main plot is divided into 1 000 bins around the circumference. The outermost blue tracks display the distribution of GC, AT, and N percentages across the bins. Scaffolds are arranged clockwise from longest to shortest and are depicted in dark grey. The longest scaffold is indicated by the red arc, and the deeper orange and pale orange arcs represent the N50 and N90 lengths. A light grey spiral at the centre shows the cumulative scaffold count on a logarithmic scale. A summary of complete, fragmented, duplicated, and missing BUSCO genes in the metazoa_odb10 set is presented at the top right. An interactive version of this figure can be accessed on the BlobToolKit viewer.

**Figure 6.**
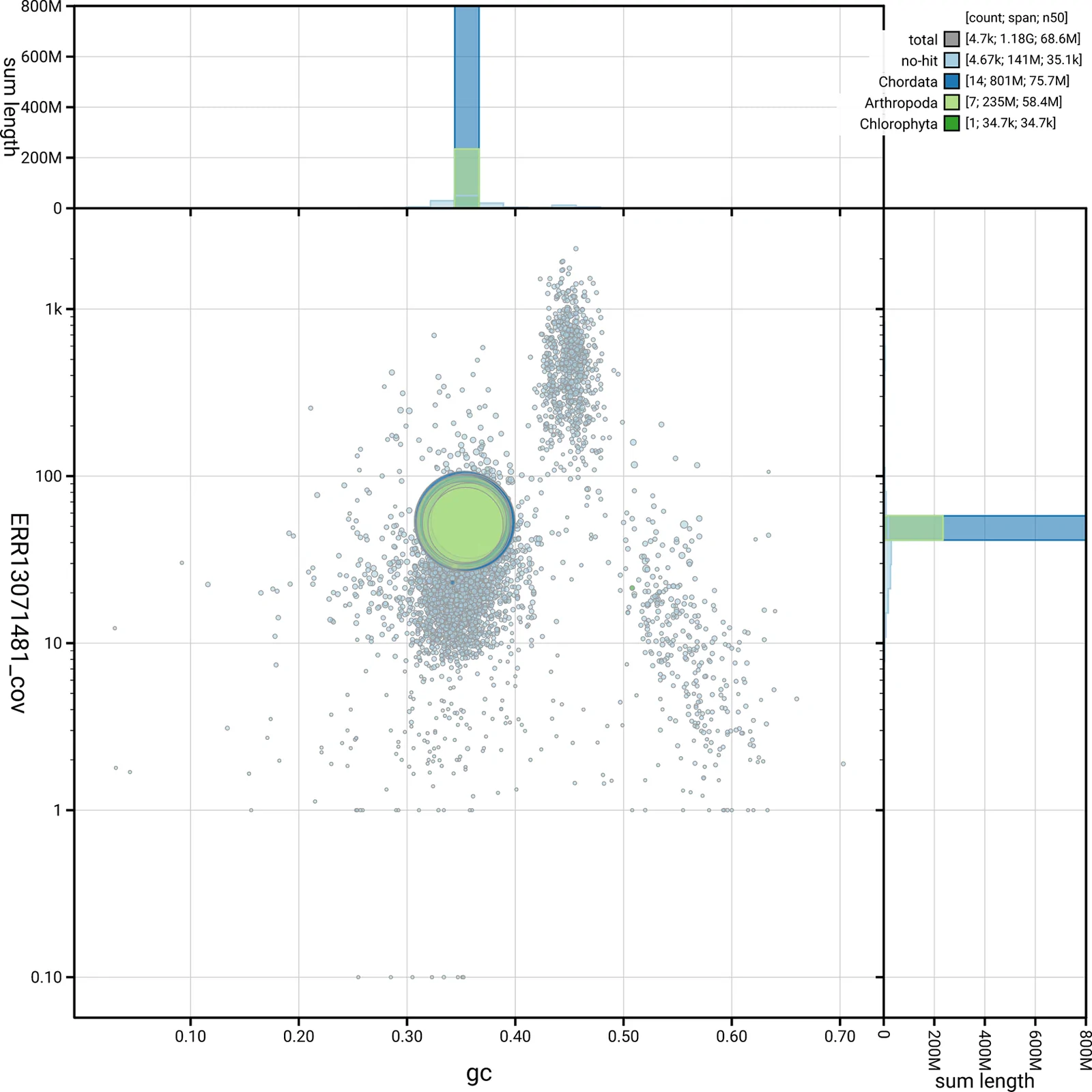
BlobToolKit blob plot for txConMacr16.3. The plot shows base coverage (vertical axis) and GC content (horizontal axis). The circles represent scaffolds, with the size proportional to scaffold length and the colour representing phylum membership. The histograms along the axes display the total length of sequences distributed across different levels of coverage and GC content. An interactive version of this figure is available on the BlobToolKit viewer.

Table 4 lists the assembly metric benchmarks adapted from Rhie *et al.* (2021) and the Earth BioGenome Project Report on Assembly Standards January 2026. The EBP metric, calculated for primary assembly, is **5.7.Q53**.

**Table 4.** Earth Biogenome Project summary metrics for the *Convolutriloba macropyga* assembly.

Measure	Value	Benchmark
EBP summary (primary)	5.7.Q53	5.C.Q40
Contig N50 length	0.19 Mb	≥ 0.1 Mb
Scaffold N50 length	68.62 Mb	= chromosome N50
Consensus quality (QV)	Primary: 53.1; alternate: 53.6; combined: 53.4	≥ 40
*k*-mer completeness	Primary: 65.15%; alternate: 56.09%; combined: 97.62%	≥ 90%
BUSCO	C:69.0% [S:65.2%, D:3.8%], F:1.8%, M:29.2%, n:954	S > 90%; D < 5%
Percentage of assembly assigned to chromosomes	88.04%	≥ 90%

**Notes:** The EBP summary uses log10(Contig N50); chromosome-level (C) or log10(Scaffold N50); Q (Merqury QV). BUSCO: C=complete; S=single-copy; D=duplicated; F=fragmented; M=missing; n=orthologues.

**Table 5.** Software versions and sources used for *Convolutriloba macropyga.*

Software	Version	Source
BLAST	2.14.0	https://ftp.ncbi.nlm.nih.gov/blast/executables/blast+/
BlobToolKit	4.3.9; 4.4.6	https://github.com/blobtoolkit/blobtoolkit
BUSCO	5.8.3; 5.5.0	https://gitlab.com/ezlab/busco
bwa-mem2	2.2.1	https://github.com/bwa-mem2/bwa-mem2
DIAMOND	2.1.8	https://github.com/bbuchfink/diamond
fasta_windows	0.2.4	https://github.com/tolkit/fasta_windows
FastK	1.1	https://github.com/thegenemyers/FASTK
GenomeScope2.0	2.0.1	https://github.com/tbenavi1/genomescope2.0
Gfastats	1.3.6	https://github.com/vgl-hub/gfastats
Hifiasm	0.19.8-r603	https://github.com/chhylp123/hifiasm
HiGlass	1.13.4	https://github.com/higlass/higlass
MerquryFK	1.1.0-c1	https://github.com/thegenemyers/MERQURY.FK
Minimap2	2.24-r1122; 2.28-r1209	https://github.com/lh3/minimap2
MitoHiFi	3.2.2	https://github.com/marcelauliano/MitoHiFi
MultiQC	1.14; 1.17 and 1.18	https://github.com/MultiQC/MultiQC
Nextflow	23.10.0; 23.10.1; 24.10.4	https://github.com/nextflow-io/nextflow
PretextSnapshot	0.0.4	https://github.com/sanger-tol/PretextSnapshot
PretextView	1.0.3	https://github.com/sanger-tol/PretextView
purge_dups	1.2.3	https://github.com/dfguan/purge_dups
samtools	1.16.1; 1.17; 1.18; 1.19.2; 1.2; 1.21; 1.6	https://github.com/samtools/samtools
sanger-tol/ascc	0.1.0	https://github.com/sanger-tol/ascc
sanger-tol/blobtoolkit	v0.8.0	https://github.com/sanger-tol/blobtoolkit
sanger-tol/curationpretext	1.4.2	https://github.com/sanger-tol/curationpretext
Seqtk	1.4-r122	https://github.com/lh3/seqtk
Singularity	3.9.0	https://github.com/sylabs/singularity
TreeVal	1.1.0	https://github.com/sanger-tol/treeval
YaHS	1.1a.2	https://github.com/c-zhou/yahs

## Author information

Contributors are listed at the following links:

• Members of the Wellcome Sanger Institute Tree of Life Management, Samples and Laboratory team
• Members of Wellcome Sanger Institute Scientific Operations – Sequencing Operations
• Members of the Wellcome Sanger Institute Tree of Life Core Informatics team
• Members of the EBI Aquatic Symbiosis Genomics Data Portal Team
• The Aquatic Symbiosis Genomics Project leadership

## Wellcome Sanger Institute – Legal and Governance

The materials that have contributed to this genome note have been supplied by a Tree of Life collaborator. The Wellcome Sanger Institute employs a process whereby due diligence is carried out proportionate to the nature of the materials themselves, and the circumstances under which they have been/are to be collected and provided for use. The purpose of this is to address and mitigate any potential legal and/or ethical implications of receipt and use of the materials as part of the research project, and to ensure that in doing so we align with best practice wherever possible.

The overarching areas of consideration are:

• Ethical review of provenance and sourcing of the material
• Legality of collection, transfer and use (national and international)

Each transfer of samples is undertaken according to a Research Collaboration Agreement or Material Transfer Agreement entered into by the Tree of Life collaborator, Genome Research Limited (operating as the Wellcome Sanger Institute) and in some circumstances other Tree of Life collaborators.

## Data Availability

European Nucleotide Archive: Convolutriloba macropyga. Accession number PRJEB75559; https://identifiers.org/ena.embl/PRJEB75559. The genome sequence is released openly for reuse. The *Convolutriloba macropyga* genome sequencing initiative is part of the Aquatic Symbiosis Genomics Project (PRJEB43743) and the Sanger Institute Tree of Life Programme (PRJEB43745). All raw sequence data and the assembly have been deposited in INSDC databases. The genome will be annotated using available RNA-Seq data and presented through the Ensembl pipeline at the European Bioinformatics Institute. Raw data and assembly accession identifiers are reported in Tables 1 and 2. Production code used in genome assembly at the WSI Tree of Life is available at https://github.com/sanger-tol . Table 5 lists software versions used in this study.
