# Supplementary material for: The chromosomal genome sequence of an acoel, Convolutriloba macropyga Shannon & Achatz, 2007 (Acoela: Convolutidae)
Source: Wellcome Open Res. 2026 Aug 20;11:533. [Version 1] doi: 10.12688/wellcomeopenres.27384.1 (PMC13650328; doi:10.12688/wellcomeopenres.27384.1)
Supplement: Peer review history

# Reviewer report: Reviewer response for version 1

**DOI:** 10.21956/wellcomeopenres.30139.r168729

**Published:** 2026-10-08

**Authors:** Ferdinand Marlétaz

**Affiliations:** 1 University College London, London, England, UK

This manuscript reports the chromosome-level genome of the photosymbiotic acoel *Convolutriloba macropyga*. Acoel genomes remain scarce, and this species is of interest for both its photosymbiosis and its asexual reproduction, so this is a valuable resource. I have a few points the authors could address.

 My main concern is the discrepancy between the k-mer-based genome size estimate (866.9 Mb in the text, 863.6 Mb in Figure 2) and the final assembly size (1,176 Mb). Combined with the very high heterozygosity (3.65%) and the low k-mer completeness of the primary assembly (65%), this raises the question of whether some haplotypic sequence remains uncollapsed. The low BUSCO duplication (3.8%) argues against this, but GenomeScope can also underestimate genome size at this level of heterozygosity. The authors should discuss this. The coverage stated (~64×) also does not match 66.51 Gb divided by either the estimated genome size or the assembly size.

 The HiFi, Hi-C and RNA-seq data come from three different specimens. As the animals are cultured and reproduce asexually, are they clonal? Given the high heterozygosity, using Hi-C from a different individual could affect scaffolding, and this should be stated.

 Given that this is an Aquatic Symbiosis Genomics project, it is surprising that the algal symbiont is not mentioned. Was symbiont sequence present in the data, and was it assembled or removed? The blob plot (Figure 6) shows a distinct cloud of small scaffolds at ~45–50% GC with much higher coverage, and only 34.7 kb is assigned to Chlorophyta. The authors should clarify what these sequences are. As the animals are fed *Artemia*, it would also be reassuring to confirm that no arthropod sequence remains, since seven chromosomes have best hits to Arthropoda (probably just the long acoel branch).

 Other points:

- The HiFi library was ultra-low-input and PCR-amplified. This probably explains the low contig N50 (0.19 Mb) and the extensive manual curation (510 breaks, 798 joins) and could be noted.
- About 12% of the assembly remains in ~4,600 unplaced scaffolds. What do these correspond to?
- BUSCO completeness is low (69.0%). How does it compare with other acoel genomes (e.g. *Hofstenia miamia*, *Symsagittifera roscoffensis*)?
- The Hi-C map shows punctate inter-chromosomal contacts at consistent positions, possibly reflecting centromere or telomere clustering. A brief comment would be interesting.
- GC% in Table 3 is rounded to 0.5%, which makes it uninformative.
- A sentence explaining why the EBP summary is 5.7.Q53 rather than 5.C.Q40 would help.

Typos, in addition to those noted by the first reviewer: "in in filtered sea water"; "sheared and purified DNA was assessed" (sentence incomplete); missing "×" in "0.85 pre-PCR clean-up", "1 clean-up", "1.0 ProNex beads" and "approximately 64 coverage".

Are sufficient details of methods and materials provided to allow replication by others?

Yes

Is the rationale for creating the dataset(s) clearly described?

Yes

Are the datasets clearly presented in a useable and accessible format?

Yes

Are the protocols appropriate and is the work technically sound?

Yes

Reviewer Expertise:

metazoan comparative genomics, evodevo, phylogenomics

I confirm that I have read this submission and believe that I have an appropriate level of expertise to confirm that it is of an acceptable scientific standard.

# Reviewer report: Reviewer response for version 1

**DOI:** 10.21956/wellcomeopenres.30139.r168725

**Published:** 2026-09-29

**Authors:** Erich M Schwarz

**Affiliations:** 1 Cornell University, Ithaca, New York, USA

Pinton et al. provide a generally sound and acceptable genome sequence analysis of the acoel Convolutriloba macropyga. I observe only a few minor points that might be good to correct, revise, or consider.

 1. In the Background, the authors write, "Because of their key phylogenetic position as the likely sister group to all other bilaterians..."  It is true that Xenacoelomorpha *may* have this position; there have been credible phylogenomic analyses supporting the Nephrozoa hypothesis (e.g., PubMed 26842059, 33741592, and 36952565). But other phylogenomic analyses have been published that instead support the phylogenetic hypothesis that Xenacoelomorpha is nested inside Deuterostomia (i.e., the Xenambulacraria hypothesis; PubMed 31104936, 36356574, and 39288803). Since this does not appear to be a fully resolved question, could the authors consider rewording or expanding this passage of their text (perhaps by adding some of the citations listed here) to make it clearer that they favor the Nephrozoa hypothesis, and that this hypothesis has exciting implications for understanding the origin of bilaterians, but that an alternative Xenambulacraria hypothesis exists?

 2. In the "Sample acquisition" section of Methods:

 2a. "adult H Convolutriloba" should read "adult Convolutriloba".

 2b. The authors write "It was collected..." after writing "A second specimen" and "a third specimen".  This is confusing; which 'it' do the authors mean?  This should be revised so that the collections of both the second and the third specimen are clearly given.

 2c. "wrtr collected" should read "were collected".

Are sufficient details of methods and materials provided to allow replication by others?

Yes

Is the rationale for creating the dataset(s) clearly described?

Yes

Are the datasets clearly presented in a useable and accessible format?

Yes

Are the protocols appropriate and is the work technically sound?

Yes

Reviewer Expertise:

Nematode genomics

I confirm that I have read this submission and believe that I have an appropriate level of expertise to confirm that it is of an acceptable scientific standard.
